# Repeated Oral Administration of Oleanolic Acid Produces Cholestatic Liver Injury in Mice

**DOI:** 10.3390/molecules18033060

**Published:** 2013-03-07

**Authors:** Yuan-Fu Lu, Xiao-Li Wan, Yasha Xu, Jie Liu

**Affiliations:** 1 Key Lab of Pharmacology, Zunyi Medical College, Zunyi 563003, China; E-Mail: xuyasha2008@163.com (Y.X.); 2 University of Kansas Medical Center, Kansas City, KS 66160, USA

**Keywords:** oleanolic acid, oral administration, cholestasis, inflammation, bile acid synthesis, bile acid transporters

## Abstract

Oleanolic acid (OA) is a triterpenoid and a fantastic molecule with many beneficial effects. However, high-doses and long-term use can produce adverse effects. This study aimed to characterize the hepatotoxic potential of OA. Mice were given OA at doses of 100–3,000 µmol/kg (45–1,350 mg/kg), po for 10 days, and the hepatotoxicity was determined by serum biochemistry, histopathology, and toxicity-related gene expression via real-time RT-PCR. Animal body weight loss was evident at OA doses of 1,000 µmol/kg and above. Serum alanine aminotransferase activities were increased in a dose-dependent manner, indicative of hepatotoxicity. Serum total bilirubin concentrations were increased, indicative of cholestasis. OA administration produced dose-dependent pathological lesions to the liver, including inflammation, hepatocellular apoptosis, necrosis, and feathery degeneration indicative of cholestasis. These lesions were evident at OA doses of 500 µmol/kg and above. Real-time RT-PCR revealed that OA produced dose-dependent increases in acute phase proteins (MT-1, Ho-1, Nrf2 and Nqo1), decreases in bile acid synthesis genes (Cyp7a1 and Cyp8b1), and decreases in liver bile acid transporters (Ntcp, Bsep, Oatp1a1, Oatp1b2, and Ostβ). Thus, the clinical use of OA and OA-type triterpenoids should balance the beneficial effects and toxicity potentials.

## 1. Introduction

Oleanolic acid (OA) is a triterpenoid that exists widely in fruits, vegetables and medicinal herbs [1,2]. OA is used as a dietary supplement and an over-the-counter Chinese medicine for the treatment of liver disorders, inflammatory diseases diabetes, and anticancer therapies in combination with other therapeutics [1,2,3,4,5]. Thus, OA and OA-type triterpenoids are emerging molecules with multiple biological effects to develop therapeutic candidates [2,3,4,5,6].

Herbal products or extracts such as OA may potentially benefit people with liver diseases by protecting against or treating experimental liver injury through antioxidant, antifibrotic, immunomodulatory, or antiviral activities [1,2,3,4,5,6]. However, like many other bioactive molecules, OA and its derivatives can also have adverse effects. For example, OA and its derivatives showed both hepatoprotective action and hepatotoxic effects towards cultured rat hepatocytes [7]. Longer-term use of OA [6,8] and OA-containing herbal mixtures [9,10] have been reported to produce side effects, even toxicity. Recently, phase-3 clinical trials of an OA derivative CDDO-Me (bardoxolone methyl) were halted (http://www.news.yahoo.com) due to safety concerns, and CDDO-Me analogues were found to produce liver injury after 3-month administration to rats [11].

The dose ranges for OA to produce liver injury are not known. This study was thus initiated to examine the hepatotoxicity potential of OA, using oral administration. The results clearly showed that “dose makes a poison”, and the hepatotoxicity produced by OA is characterized by cholestasis associated with alterations in liver transporters and bile acid-metabolism genes. 

## 2. Results and Discussion

### 2.1. Dose-Response of Liver Injury Produced by OA

#### 2.1.1. Animal Body Weight and Liver Weight

OA is often given via injections to examine its beneficial effects [1,2,3,4,5,6]. Due to its poor solubility, most of the studies used OA suspensions in 2% Tween-80 saline for injection [12]. Considering that OA is an oral drug, this study was thus initiated to examine oral hepatotoxic potential of OA. OA was dissolved/suspended in corn oil and was gavaged to Kunming (KM) mice, a mouse strain widely used in China for pharmacology and toxicology studies. 

**Figure 1:**
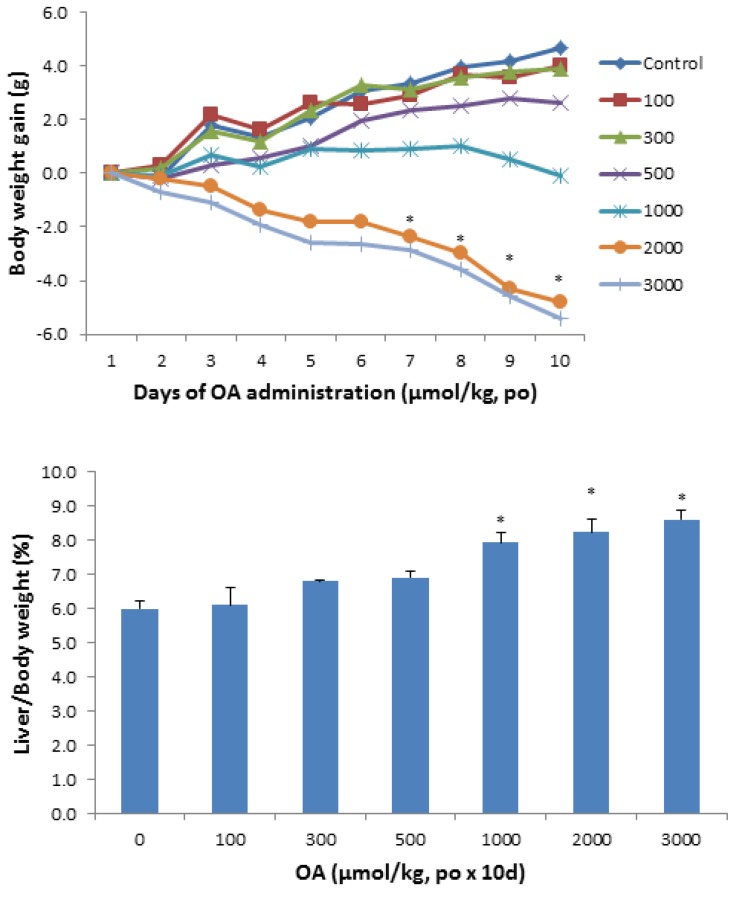
Effect of oral OA on body weight gain and liver index. Mice were gavaged OA at doses of 100–3,000 µmol/kg (equal to 45–1,350 mg/kg), once daily for 10 days. Animal body weights were recorded daily and the liver weights were recorded at the end of experiments. Data are mean ± SEM (n = 5–8). *Significantly different from control, *p* < 0.05.

Figure 1 shows the animal body weight and liver weight changes following OA administration in KM mice. Mice were given OA (100–3,000 µmol/kg), daily for 10 days. All mice survived the doses used in the study, but at doses of 1,000 µmol/kg and above, body weight loss was evident. At the highest dose of OA, the loss of body weight approached 20%. The liver/body weight ratios increased to 35% at the highest dose of OA.

#### 2.1.2. Serum Biochemistry

**Figure 2:**
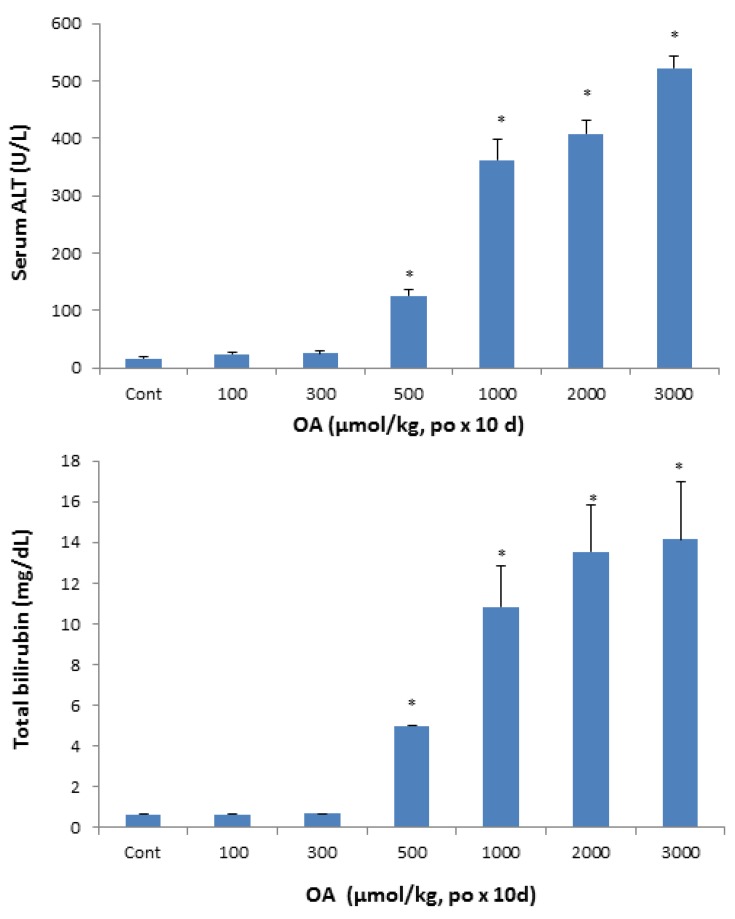
Effect of oral OA on serum biochemistry. Mice were gavaged OA at doses of 100-3,000 µmol/kg (equal to 45-1350 mg/kg), once daily for 10 days. Twenty-four hours after the last dose, blood was collected to determine changes in serum activities of alanine aminotransferase (ALT) and the concentration of total bilirubin as detailed in Methods. Data are mean ± SEM (n = 5–8). *Significantly different from control, *p* < 0.05.

At the end of experimentation, blood was collected to separate serum and serum biochemistry was performed. A dose-dependent increase in serum activities of alanine aminotransferase (ALT) was remarkable, up to 20-fold (Figure 2, top), indicative of hepatocellular damage and cell death at OA doses of 500 µmol/kg and above. Serum AST showed the similar results (data not shown). Serum total bilirubin concentrations, a biomarker for cholestasis, increased at doses of 500 µmol/kg and above, and reached 13 mg/dL as compared to 0.8 mg/dL in controls. 

#### 2.1.3. Histopathology

**Figure 3:**
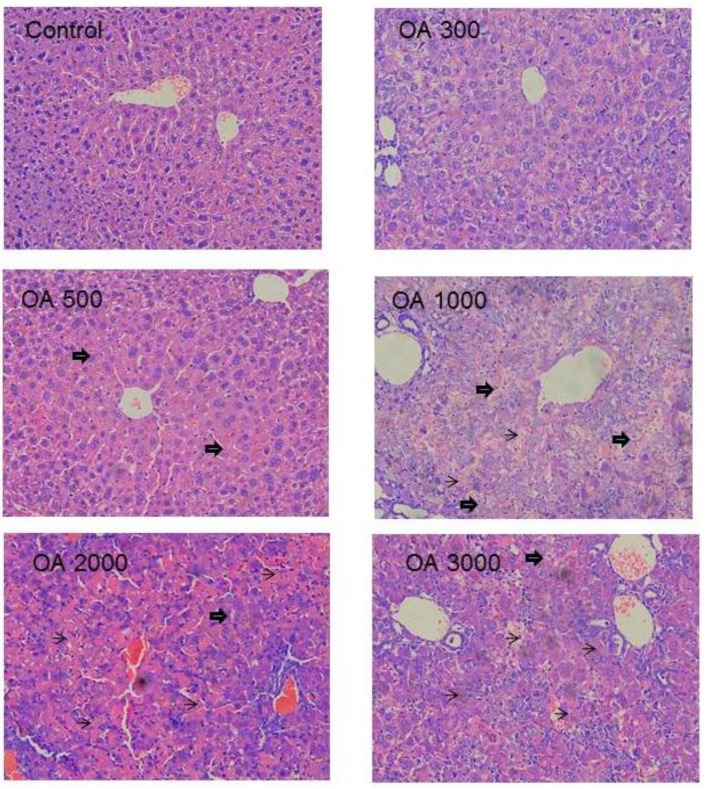
Representative liver histopathology. Mice were gavaged OA at doses of 100–3,000 µmol/kg (equal to 45–1,350 mg/kg) for 10 days. At the end of experiment, livers were fixed in neutral formalin and subjected to standard histology procures. Slides were stained H&E. big-arrows indicate feathery degeneration indicative of cholestasis and thin-arrows indicate hepatocellular cell death. Magnitude (×200).

Histopathology was in a good agreement with serum biochemistry. At the therapeutic dose of 100–300 µmol/kg, the liver morphology was normal. At 500 µmol/kg of OA, foci of liver degeneration could be seen, but the liver morphology was mainly normal. At 1,000 µmol/kg of OA, cholestatic liver injury was evident, as evidenced by the feathery-like degeneration and hepatocellular death, and at the highest dose of 3,000 µmol/kg, widespread cell death was evident (Figure 3).

### 2.2. Effects of OA on Acute-Phase Protein Genes and Inflammatory Markers

**Figure 4:**
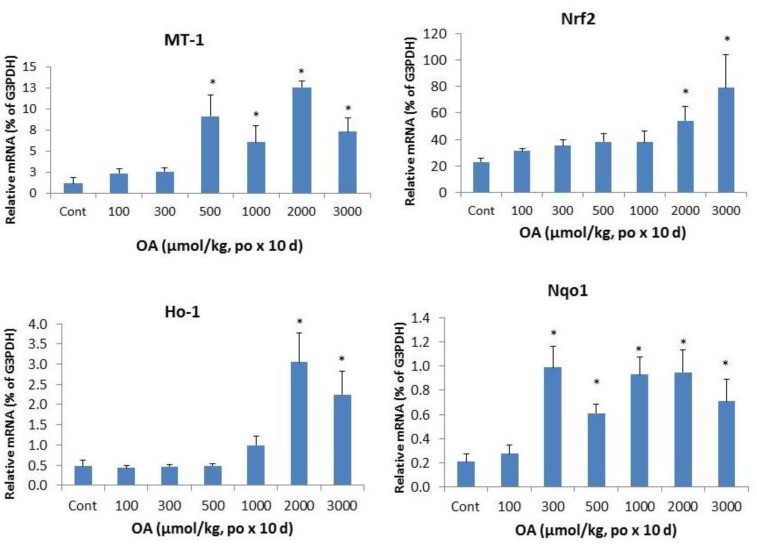
Expression of inflammatory and stress markers. Mice were gavaged OA at doses of 100–3,000 µmol/kg (equal to 45–1,350 mg/kg). Twenty-four hours after the last dose, Livers were collected to isolate total RNA. The expression of metallothionein-1 (MT-1), heme oxygenase-1 (Ho-1), Nrf2, and NADH quinone oxidase -1 (Nqo1) were determined by real-time RT-PCR as detailed in the Methods. Data are mean ± SEM (n = 5–8). *Significantly different from control, *p* < 0.05.

Inflammation is a hallmark of cholestatic liver injury [13,14,15,16,17]. Metallothionein (MT) and heme oxygenase 1 (Ho-1) are acute phase protein genes sensitive to liver injury. The left panel of Figure 4 shows the dramatic induction of MT-1 at OA doses of 500 µmol/kg and above, and induction of Ho-1 at doses of OA 1,000 µmol/kg and above, indicative of acute stress to the liver. Nrf2 and its target gene Nqo1 were also induced. The apparent induction of Nrf2 was evident at OA 2,000 µmol/kg and above, but the induction of Nrf2-target gene Nqo1 was more sensitive at OA 300 µmol/kg and above, with approximately 5-fold induction was observed.

### 2.3. Effects of OA on Bile Acid Metabolism Genes

**Figure 5:**
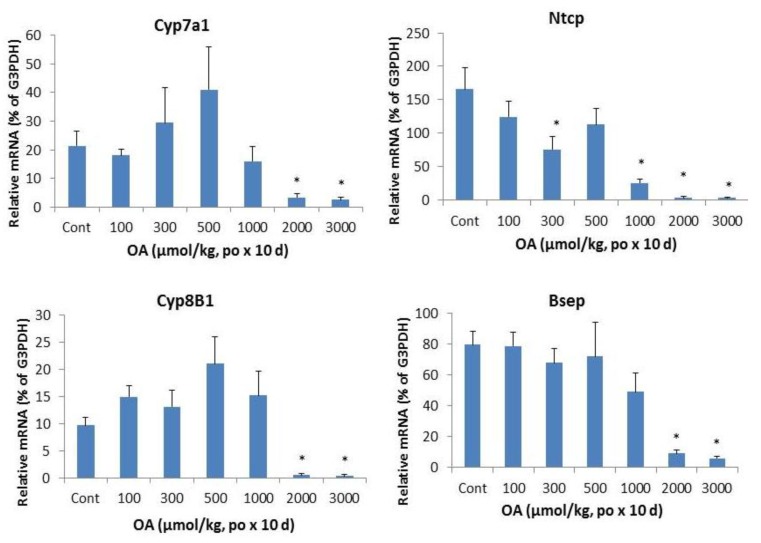
Effect of oral OA on expression of bile acid synthesis and transport genes. Mice were gavaged OA at doses of 100–3,000 µmol/kg (equal to 45–1,350 mg/kg). Twenty-four hours after the last dose, Livers were collected to isolate total RNA. The expression of hepatic transport genes were determined by real-time RT-PCR as detailed in the Methods. Data are mean ± SEM (n = 5–8).*Significantly different from control, *p* < 0.05.

Figure 5 illustrates the effects of OA on bile acid metabolism gene expressions. The bile acid synthesis rate-limiting enzyme Cyp7a1 was dramatically decreased by OA. The classic bile acid synthesis pathway gene Cyp8b1 was also suppressed. 

In liver, the bile acid uptake transporters located on the basolateral membranes of hepatocytes include the liver specific bile acid transporter Na^+^-taurochlorate co-transporting ploypeptide (Ntcp), while canalicular transporter responsible for biliary excretion of bile acids is bile salt export pump (Bsep). Both Ntcp and Bsep were decreased at OA 1,000 µmol/kg and above.

### 2.4. Effects of OA on Hepatic Transporters

In liver, organic anion transporting peptide (Oatp)1a1 [18,19,20] and 1b2 play important roles in drug transport and bile acid uptake [20,21]. OA treatment resulted in a marked decrease in mRNA levels of Oatp1a1 and Oatp1b2. OA also showed biphasic effects on hepatic Ostβ: at the doses of 300–2,000 µmol/kg, OA increased the mRNA levels of Ostβ, but at the higher doses, no increase was evident, probably due to the liver toxicity and hepatocellular death. 

### 2.5. Discussion

The present study clearly demonstrated that oral OA administration produced hepatotoxicity characterized by cholestasis in outbred KM mice in a dose-dependent manner. This study is in good agreement with cholestasis produced by repeated OA injections [22], in which the hepatotoxicity occurred after 200 µmol OA (90 mg/kg) and the oral dose was 3–5 fold higher than the injection dose (500 µmol/225 mg/kg *vs.* 100–200 µmol/kg). The margin of oral OA safety is also narrow, as the toxic dose (TD, 500 µmol/kg)/effective dose (ED, 100–200 µmol/kg) in KM mice is less than 5. Clinically, the dose of OA can be used as high as 80 mg, three times per day (240 mg/person/day) for months [23]. Thus, caution should be taken when longer-term use of this molecule in the treatment of human diseases.

Herbal hepatotoxicity is an expanding but poorly defined problem as herbal medicines become more popular in the developing and industrialized countries [24,25,26]. Herbal toxicity could be produced by herbal mixtures, single herbs, or bioactive molecules such as OA [6,7,8,9,10,21,22,23]. For example, the “hepatoprotective” herbs or molecules such as rhubarb, have been documented as having both therapeutic and toxic effects, leading to the complex problem of distinguishing the benefits from the risks [24]. Therefore, the beneficial effects of OA and OA-type triterpenoids should be balanced between hepatoprotective action and hepatotoxicity. 

Histopathology clearly shows the intrahepatic cholestasis produced by OA at doses greater than 500 µmol/kg. The feathery-like hepatocyte degeneration and hepatocellular death are apparent (Figure 3), corresponding to increased serum total bilirubin and increased serum ALT (Figure 2). Feathery-like degeneration occurred when bile droplets fill in the hepatocytes, an indication of cholestatic liver injury [27].

Cholestasis results in a buildup of bile acids in serum and in hepatocytes and is associated with inflammatory responses [13]. Mechanisms of cholestatic liver injury include bile acid-induced apoptosis, bile acid-induced aberrant cell signaling and the inflammatory responses in the pathophysiology [14]. Bile stagnation causes the increases in Zn, alone with the Zn-binding protein MT [22], making induction of MT as an early sign of cholestic liver injury [14,15]. Heme oxygenase-1 (Ho-1) overexpression is increased after bile duct ligation in rats [16]. During cholestasis, nuclear factor-E2-related factor 2 (Nrf2) expression in liver is critical for induction of NAD(P)H:quinone oxidoreductase 1 (Nqo1), in an attempt to fight against increased oxidative stress during cholestasis [17].

Consistent with the cholestasis produced by OA, the expression of genes encoding bile acid metabolism was altered. The “classic” bile acid synthesis pathway (Cyp7a1 and Cyp8b1) [18] was decreased at higher doses of OA. The hepatic uptake transporter Ntcp [19] was suppressed, corresponding to increased serum bile acids concentrations. The primary transporter responsible for bile salt secretion is Bsep (Abcb11), a member of the ATP-binding cassette (ABC) superfamily, which is located at the bile canalicular apical domain of hepatocytes [20]. OA also decreased the mRNA levels of Bsep at higher doses. OA is a molecule capable of inducing intrahepatic cholestais with disruption of bile acid homeostasis genes.

The expression of uptake transporters, including Oatp1a1, Oatp1a4, Oatp1b2, and Oatp2b1 is important for hepatic uptake xenobiotics [21], and plays important role in the body defense against toxicants [28,29]. In the present study, expressions of Oatp1b2 and Oatps were suppressed, even at the dose without producing apparent liver injury (100 µmol/kg). Oatp1b2 plays an important role in hepatocellular uptake of xenobiotics and bile acids [17,20], and mediates the hepatocellular uptake of phalloidin and other toxicants [20,21,28,29]. In the present study, both Oatp1a1 and Oatp1b2 were dramatically suppressed, suggesting that down-regulation of Oatp transporters could be a cause for the increased serum bile acid concentrations as the liver failed to take bile acids back into the liver for metabolism.

**Figure 6:**
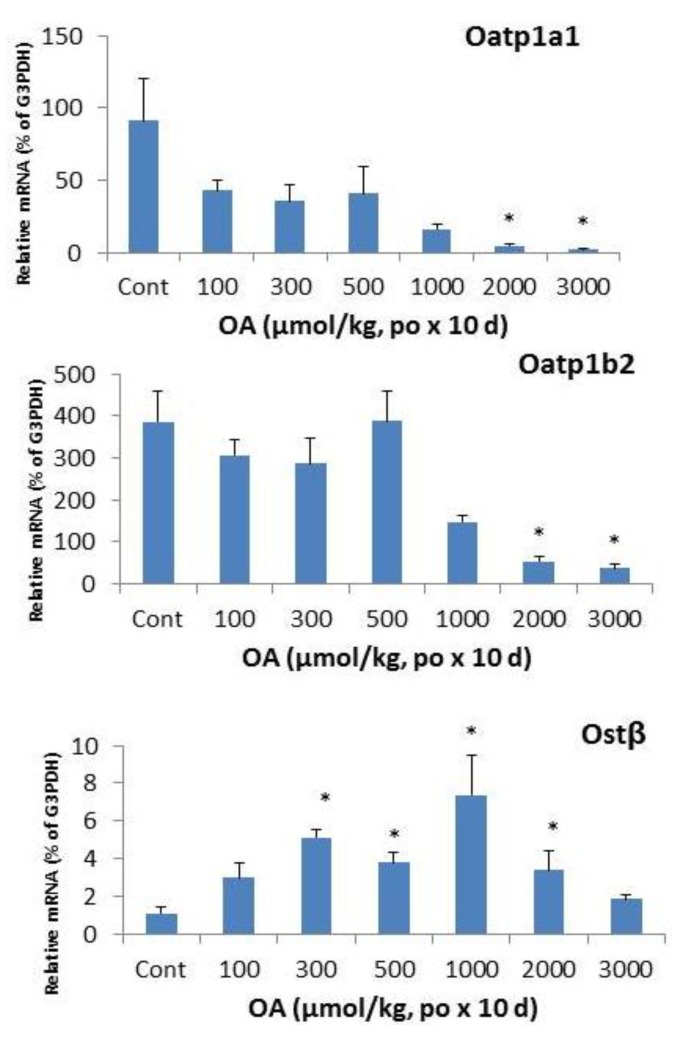
Expression of hepatic transporters. Mice were gavaged OA at doses of 100–3,000 µmol/kg (equal to 45–1,350 mg/kg). Twenty-four hours after the last dose, Livers were collected to isolate total RNA. The expression of hepatic transport Oatp1a1, Oatp1b2, and Ostβ were determined by real-time RT-PCR as detailed in the Methods. Data are mean ± SEM (n = 5–8). *Significantly different from control, *p* < 0.05.

The Ostα/Ostβ transporter is thought to be partially responsible for the efflux of bile acids back into the blood [30]. The mRNA expression of the two genes is increased in patients with primary biliary cirrhosis (PBC), as well as in bile-duct ligation (BDL) rats [30]. In FXR-null mice after BDL, the mRNA expression of Ostα and Ostβ did not increase, suggesting that the induction of Ostα/Ostβ by bile acids is FXR dependent [30]. OA decreased Ostα but increased Ostβ after repeated sc injections [22]. Following oral OA, Ostβ expression was also increased and reached 7-folds at the dose of 1000 µmol/kg, but decreased at higher doses, probably due to liver damage (Figure 6). 

## 3. Experimental

### 3.1. Chemicals

Oleanolic acid (OA, purity >98) was obtained from Guiyang Pharmaceutical Company as described previously [12]. All other chemicals were reagent grade.

### 3.2. Animals and Treatments

Adult male outbred Kunming (KM) mice were obtained from the Animal Breeding Center (Chongqing, China), and maintained in the SPF-grade animal facilities. Mice were acclimated for 2 weeks in a temperature-humidity controlled facility with a standard 12-h light schedule. Mice had free access to standard rodent chow and tap water. All animal procedures followed the NIH Guide for Humane Use and Care Animals, and approved by Institutional Animal Use and Care Committee of Zunyi Medical College. Mice were treated with OA (100–3,000 μmol/kg, *i.e.*, 45–1,350 mg/kg, dissolved/suspended in corn oil, po) once daily for 10 consecutive days. Control mice received the same volume of corn oil. Twenty-four-hours after the last dose, mice were anesthetized with pentobarbital (50 mg/kg, ip), and blood and livers were collected for analysis.

### 3.3. Serum Biochemistry

Blood was placed on ice for 60 min and centrifuged to separate serum. Serum samples were analyzed by standard enzymatic assays using commercial kits for alanine aminotransferase (ALT), aspartate aminotransferase (AST), direct and total bilirubin (DBIL and TBIL) in accordance with the manufacturer’s protocols (Jiang-Cheng Biological, Nanjin, China).

### 3.4. Histopathology

Liver samples were fixed in 10% formalin, and subjected to standard histological procedures and paraffin embedding. Liver sections (5 μm in thickness) were stained with hematoxylin and eosin and evaluated for histopathological lesions.

### 3.5. RNA Isolation

Approximate 50–100 mg of liver was homogenized in 1 mL Trizol (TakaRa Biotechnology, Dalian, China). Total RNA was extracted according to the manufacturer’s instructions, followed by purification with Total RNA (Mini) Kit (Watson Biotechnology, Shanghai, China). The quality and quantity of RNA was determined by the 260/280 ratios.

### 3.6. Real-Time RT-PCR Analysis

Total RNA was reverse transcribed with Multiscript reverse transcriptase using a High Capacity RT kits from Applied Biosystems (Applied Biosystems, Foster City, CA, USA). Primers were designed with Primer3 software (version 4), and listed in Table 1. The Power SYBR Green Master Mix (Applied Biosystems) was used for real-time RT-PCR analysis. Differences in gene expression between groups were calculated using cycle threshold (Ct) values, which were normalized with G3PDH, and expressed as relative transcript levels, setting controls as 100%.

### 3.7. Statistical Analysis

All data were analyzed using a one-way ANOVA, followed by a Duncan’s multiple range *post hoc* test. Significance was set at *p* < 0.05. Bars represent means ± SEM.

**Table 1:** Real-time RT PCR primer sequences.

Gene	GenBank#	Forward	Reverse
*Besp*	NM_021022	GGACAATGATGTGCTTGTGG	CACACAAAGCCCCTACCAGT
*Cyp7a1*	NM_007824	ATCCTGGCAAACAGAAATCG	GGCCAAGTCTGGTTTCTCTG
*Cyp8b1*	NM_010012	AGTTGCAGCGTCTCTTCCAT	CCTTGCTCCCTCAGAAACTG
*G3PDH*	M32599	AACTTTGGCATTGTGGAAGG	GGATGCAGGGATGATGTTCT
*HO-1*	M33203	CCTCACTGGCAGGAAATCATC	CCTCGTGGAGACGCTTTACATA
*MT-1*	NM_013602	CTCCGTAGCTCCAGCTTCAC	AGGAGCAGCAGCTCTTCTTG
*Nqo1*	BC004579	TATCCTTCCGAGTCATCTCTAGCA	TCTGCAGCTTCCAGCTTCTTG
*Nrf2*	BC026943	CGAGATATACGCAGGAGAGGTAAGA	GCTCGACAATGTTCTCCAGCTT
*Ntcp*	NM_011387	GGTGCCCTACAAAGGCATTA	ACAGCCACAGAGAGGGAGAA
*Oatp1a1*	NM_013797	ATCCAGTGTGTGGGGACAAT	GCAGCTGCAATTTTGAAACA
*Oatp1b2*	NM_020495	CAAACTCAGCATCCAAGCAA	GGCTGCCAAAAATATCCTGA
*Ostâ*	NM_178933	ATCCTGGCAAACAGAAATCG	GGCCAAGTCTGGTTTCTCTG

## 4. Conclusions

In summary, the present study clearly demonstrated that repeated oral OA administration produced cholestatic liver injuries in outbred KM mice, illustrating the hepatotoxic potential of a presumed hepatoprotective compound. The mechanism of cholestasis liver injury may result from the down-regulation of hepatic transporters, disruption of bile acid uptake and metabolism, resulting in bile acid-induced liver injury. Thus, caution should be taken when using OA and OA-type triterpenoids for a longer term to treat liver and other disorders.
